# The association between systemic immune-inflammation index and post-stroke depression: a meta-analysis

**DOI:** 10.3389/fpsyt.2026.1817964

**Published:** 2026-06-05

**Authors:** Wendi Zhang, Geng Chang, Zhen Mu

**Affiliations:** 1 Department of Neurology, The First Affiliated Hospital of Dalian Medical University, Dalian, China; 2 Dalian Medical University, Dalian, China

**Keywords:** depression, meta-analysis, risk factor, stroke, systemic immune-inflammation index

## Abstract

**Background:**

Post-stroke depression (PSD) is a common neuropsychiatric complication that adversely affects recovery and prognosis after stroke. The systemic immune-inflammation index (SII), a composite biomarker derived from peripheral blood counts, reflects systemic inflammatory status and has been associated with adverse neurological outcomes. However, the relationship between SII and PSD remains uncertain. We conducted a meta-analysis to clarify this association.

**Methods:**

PubMed, Embase, Web of Science, Wanfang, and CNKI were searched for observational studies evaluating the association between SII and PSD in adult stroke patients. Odds ratios (ORs) with 95% confidence intervals (CIs) were pooled using a random-effects model by incorporating the potential influence of heterogeneity.

**Results:**

Seven studies involving 2,780 patients were included, among whom 822 developed PSD. Compared with lower SII levels, high SII at admission was significantly associated with an increased risk of PSD (OR = 2.14, 95% CI: 1.74–2.64; I² = 22%). Sensitivity analyses by excluding one study at a time showed similar results (pooled OR range: 2.06–2.38, all *p*-values < 0.05), which confirmed the stability of the findings. The association remained consistent across study design, stroke type, age, sex proportion, SII cutoff method, cutoff value, PSD assessment tool, and study quality (all *p* for subgroup differences > 0.05).

**Conclusions:**

Elevated SII may be independently associated with an approximately twofold increased risk of PSD. SII may serve as a simple and accessible inflammatory biomarker for early identification of patients at higher risk of developing PSD.

**Systematic Review Registration:**

https://www.crd.york.ac.uk/prospero/, identifier CRD420261326749.

## Introduction

Post-stroke depression (PSD) is one of the most common neuropsychiatric complications after stroke, affecting approximately 20–35% of survivors within the first year (1, 2). PSD is associated with impaired functional recovery (3), reduced quality of life (4), poorer cognitive outcomes (5), increased healthcare utilization (6), and higher long-term mortality (7). Established risk factors include female sex, prior history of depression, greater stroke severity, physical disability, cognitive impairment, social isolation, and vascular comorbidities (1, 8). However, these clinical predictors do not fully explain the occurrence of PSD, and reliable, objective biomarkers for early risk stratification remain limited (9). Increasing evidence suggests that inflammation plays a pivotal role in the pathophysiology of depression after stroke, through mechanisms involving neuroinflammation, blood–brain barrier disruption, dysregulation of monoamine neurotransmitters, and activation of the hypothalamic–pituitary–adrenal axis (10, 11). Therefore, identifying novel inflammation-related biomarkers that are simple, accessible, and clinically applicable may improve early detection and targeted intervention for PSD.

The systemic immune-inflammation index (SII), calculated as platelet count × neutrophil count divided by lymphocyte count, is a composite marker reflecting the balance between innate immune activation and adaptive immune regulation (12). Elevated SII indicates enhanced neutrophil- and platelet-mediated inflammatory responses together with relative lymphopenia, suggesting systemic immune dysregulation (13, 14). Experimental and clinical studies have demonstrated that systemic inflammation contributes to depressive symptoms by promoting proinflammatory cytokine release, altering serotonergic and glutamatergic signaling, and inducing microglial activation (15, 16). SII has been associated with adverse outcomes in cardiovascular and cerebrovascular diseases and has been linked to poor functional recovery and increased mortality after stroke (17). However, although several observational studies have explored the association between SII and PSD, their findings have been inconsistent and limited by small sample sizes and heterogeneous methodologies (18–24). To date, no comprehensive synthesis has quantitatively evaluated this relationship. Therefore, we conducted a systematic review and meta-analysis to clarify the association between SII and the risk of PSD in patients with stroke and to assess the robustness and consistency of the available evidence.

## Methods

The meta-analysis was carried out in accordance with established methodological guidance, following the principles outlined in the PRISMA 2020 statement (25) and the Cochrane Handbook for Systematic Reviews and Meta-Analyses (26), encompassing protocol planning, study selection, data extraction, statistical analysis, and reporting. The study protocol was registered prospectively in the PROSPERO database (registration number: CRD420261326749).

### Database search

We carried out a comprehensive literature search across PubMed, Embase, Web of Science, Wanfang, and China National Knowledge Infrastructure (CNKI) to identify eligible studies for inclusion. The search strategy was constructed using the combination of the following terms: (1) “systemic inflammation index” OR “systemic immune-inflammation index” OR “SII” OR “systemic immune-inflammatory index” OR “systemic-immune-inflammation index”; (2) “stroke” OR “cerebral infarction” OR “cerebral hemorrhage” OR “cerebrovascular accident” OR “intracranial hemorrhage”; and (3) “depression” OR “depressive”. Only full-text, peer-reviewed articles published in English or Chinese and conducted in human populations were considered eligible. We also manually examined the reference lists of relevant reviews and original studies to capture additional potentially eligible reports. Each database was searched from inception through January 12, 2026. The complete search strategies for all databases are provided in **Supplementary File 1**.

### Study inclusion and exclusion criteria

The selection of studies was guided by the PICOS principle:

P (Population): Adult patients (≥18 years) with a confirmed diagnosis of stroke (ischemic stroke, hemorrhagic stroke, or mixed types).
I (Exposure): Studies were required to report baseline SII measured in peripheral blood, calculated as platelet count × neutrophil count/lymphocyte count. SII had to be categorized into high versus low groups according to predefined cutoff values (e.g., median, tertiles, quartiles, or ROC-derived thresholds). A high SII at baseline, as defined by the cutoff values of the original studies, was considered as exposure.
C (Comparator): The comparator consisted of patients with lower SII levels as defined by each individual study.
O (Outcome): The outcome of interest was PSD, diagnosed using standardized diagnostic criteria, including the Diagnostic and Statistical Manual of Mental Disorders (DSM) or the International Classification of Diseases (ICD), or assessed using validated depression rating scales with predefined cutoff values, such as the Hamilton Depression Rating Scale (HAMD), the Patient Health Questionnaire-9 (PHQ-9), the Beck Depression Inventory (BDI), or the Hospital Anxiety and Depression Scale-Depression subscale (HADS-D).
S (Study design): Eligible studies included prospective cohort studies, retrospective cohort studies, and case–control studies published as full-text articles in peer-reviewed journals.

Studies were excluded if they were reviews, meta-analyses, editorials, letters, case reports, or case series; if they included patients without confirmed stroke or did not clearly define PSD; if SII was not reported or not categorized into high and low groups; if only other inflammatory markers were evaluated without separate SII data; if effect estimates were not reported and could not be calculated; if duplicate or overlapping populations were identified (in which case the study with the largest or most complete dataset was included); if they were conducted in animals; or if they exclusively involved pediatric populations.

### Study quality evaluation and data extraction

Two reviewers independently performed the literature search, screened eligible studies, assessed study quality, and extracted relevant data. Any disagreements were resolved through discussion, and when necessary, by consulting the corresponding author. Study quality was appraised using the Newcastle–Ottawa Scale (NOS) (27). The NOS examines methodological rigor across selection, comparability, and outcome ascertainment domains. Total scores vary from 0 to 9, and studies achieving ≥ 8 points were considered to be of high quality. Extracted data included study characteristics (first author, publication year, country, and study design), patient characteristics (numbers of included patients, diagnosis, mean age, and sex distribution), exposure assessment (timing of SII measurement, methods for determining the cutoffs of SII, and cutoff values for defining a high SII in each study), follow-up durations, outcome validation (methods for the diagnosis of PSD and number of patients who developed PSD during follow-up), and covariates adjusted when the association between a high SII and PSD was analyzed.

### Statistical analyses

The association between SII and PSD was evaluated by combining odds ratios (ORs) and their corresponding 95% confidence intervals (CIs), compared between patients with a high vs. a low SII at baseline. When necessary, effect estimates and standard errors were derived from reported 95% CIs or *p* values. All estimates were log-transformed before pooling to enhance normal distribution assumptions and stabilize variances (26). To evaluate variability across studies, we applied the Cochrane Q test and calculated the I² statistic (28). I² values below 25% were classified as low heterogeneity, 25–75% as moderate, and above 75% as high heterogeneity. Pooled effect estimates were calculated using a random-effects model to accommodate variability across studies (26). We performed leave-one-out sensitivity analyses, sequentially excluding individual studies to assess the robustness of the findings (29). To identify possible sources of between-study variability, we performed predefined subgroup analyses stratified by study design (prospective vs. retrospective), diagnosis (ischemic vs. mixed stroke types), mean age of the participants, proportions of men, methods for determining the cutoffs of SII, cutoff values for defining a high SII, methods for the diagnosis of PSD, and NOS scores of the included studies. The medians of the continuous variables were used as cutoff values to define subgroups in order to ensure a balanced distribution of studies across each subgroup. To assess potential publication bias, we inspected funnel plots for asymmetry and conducted Egger’s regression analysis (30). A two-tailed *p* value < 0.05 was considered statistically significant. All statistical analyses were performed using RevMan (version 5.3; Cochrane Collaboration, Oxford, UK) and Stata (version 17.0; StataCorp, College Station, TX, USA).

## Results

### Database search results

**Figure 1:**
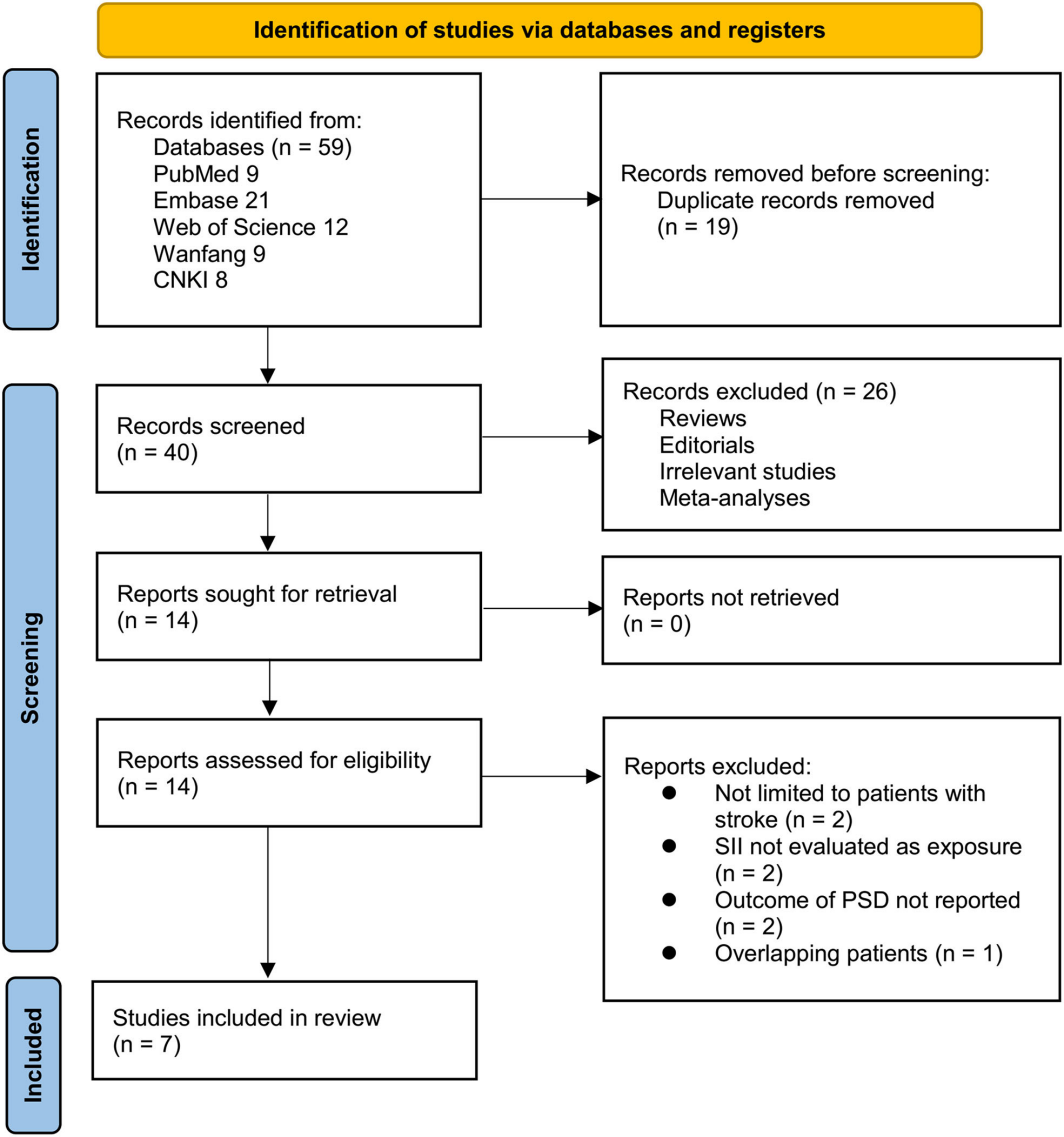
Flow diagram of the study selection process.

The study selection procedure is illustrated in **Figure 1**. A total of 59 records were retrieved from the five databases, and 19 duplicates were removed. Following screening of titles and abstracts, 26 records were excluded for failing to meet the predefined inclusion criteria. Fourteen articles underwent full-text evaluation by two independent reviewers, after which seven studies were excluded for the reasons detailed in **Figure 1**. Ultimately, seven studies met the eligibility criteria and were included in the quantitative meta-analysis (18–24).

### Overview of study characteristics

The characteristics of the included studies are summarized in **Table 1**. A total of seven observational studies published between 2021 and 2025 were included, comprising three prospective studies (18, 20, 22) and four retrospective studies (19, 21, 23, 24). All the studies were conducted in China (Wenzhou, Baoji, Chongqing, Yichang, Sanmenxia, Qingdao, and Guangzhou) (18–24). Overall, a total of 2,780 patients with stroke were included in the meta-analysis, with five studies including only patients with ischemic stroke (18–20, 22, 24), and the other two studies (21, 23) including both ischemic and hemorrhagic stroke. The mean age of participants ranged from 48.4 to 68.4 years, and the proportion of men varied between 46.7% and 79.6%. In all studies, SII was measured at admission. The methods used to define SII cutoffs included tertile comparison (18, 19, 22), quartile comparison (21), and median-based categorization (20, 23, 24), with cutoff values ranging from 449.7 to 645.6. Follow-up duration ranged from 0.5 months to 12 months. PSD was diagnosed using the HAMD in six studies (18–20, 22–24) and the PHQ-9 in one study (21). Accordingly, a total of 822 patients were diagnosed with PSD during follow-up. All the included studies performed multivariable adjustment, commonly controlling for age and sex, and most additionally adjusted for stroke severity (e.g., NIHSS score), vascular risk factors (e.g., hypertension, diabetes mellitus, coronary heart disease), lifestyle factors, and socioeconomic indicators to varying degrees.

**Table 1:** Characteristics of the included studies.

Study	Country	Design	No. of patients included	Stroke type	Mean age (years)	Men (%)	Timing of SII measuring	Methods for determining the cutoff of SII	Cutoff value of SII	Follow-up duration (months)	Methods for the diagnosis of PSD	Number of patients with PSD	Variables adjusted
Hu 2021 (18)	Wenzhou, China	Prospective	423	Ischemic	62.6	64.3	At admission	T3:T1	547.3	1	HAMD	129	Age, sex, education years, baseline NIHSS score, and CHD
Luo 2024 (19)	Baoji, China	Retrospective	307	Ischemic	61.0	51.8	At admission	T3:T1	501.0	1	HAMD	103	Age, sex, education years, NIHSS score, and mRS score
Sun 2024 (20)	Chongqing, China	Prospective	427	Ischemic	58.0	79.6	At admission	Median	554.7	12	HAMD	144	Age, sex, baseline NIHSS, current smoking, current drinking, DM, hypertension, hyperlipidemia, previous CHD, previous stroke, BMI, education levels, and physical activity
Xiao 2025 (24)	Yichang, China	Retrospective	332	Ischemic	63.1	46.7	At admission	Median	449.7	0.5	HAMD	88	Age, sex, CRP, living alone, hypertension, Hcy, Education levels, and NIHSS score
Wu 2025 (23)	Sanmenxia, China	Retrospective	260	Mixed	68.4	74.2	At admission	Median	508.2	1	HAMD	97	Age, sex, DM, monthly disposable medical expenses, left hemisphere lesion, NIHSS score, and Hcy
Su 2025 (22)	Qingdao, China	Prospective	318	Ischemic	63.0	68.2	At admission	T3:T1	645.6	3	HAMD	98	Age, sex, marital status, education, current smoking, current alcohol drinking, hypertension, DM, hyperlipidemia, CHD, AF, baseline NIHSS score, Barthel Index score, mRS score, lesion location, and Hcy
Li 2025 (21)	Guangzhou, China	Retrospective	713	Mixed	48.4	48.7	At admission	Q4:Q1	592.8	1	PHQ-9	163	Age, sex, marital status, family income level, smoking history, drinking history, and CHD

SII, systemic immune-inflammation index; PSD, post-stroke depression; HAMD, Hamilton Depression Rating Scale; PHQ-9, Patient Health Questionnaire-9; NIHSS, National Institutes of Health Stroke Scale; mRS, modified Rankin Scale; CHD, coronary heart disease; CHF, congestive heart failure; DM, diabetes mellitus; BMI, body mass index; CRP, C-reactive protein; Hcy, homocysteine; AF, atrial fibrillation; Q4, fourth quartile; Q1, first quartile; T3, third tertile; T1, first tertile.

### Study quality evaluation

Methodological quality was assessed using the NOS, and detailed results are presented in **Table 2**. The NOS scores ranged from 7 to 9, indicating overall moderate to high methodological quality. Two studies (20, 22) achieved the maximum score of 9, reflecting strong cohort representativeness, appropriate selection of comparison groups, reliable exposure ascertainment, adequate control for confounders, sufficient follow-up duration, and complete outcome assessment. Three studies (18, 19, 23) scored 8, primarily due to limitations in follow-up duration. The remaining two studies (21, 24) scored 7, mainly attributable to limited representativeness of the exposed cohort and shorter follow-up periods. Importantly, all studies received full stars for exposure ascertainment, confirmation that PSD was not present at baseline, assessment of outcomes, and adequacy of follow-up. All included studies controlled for age and sex, and most additionally adjusted for other important confounding factors, including stroke severity and vascular comorbidities. Overall, the methodological quality of the included studies was considered moderate to high, supporting the reliability of the pooled estimates evaluating the association between SII and PSD.

**Table 2:** Study quality evaluation via the Newcastle-Ottawa scale.

Study	Representativeness of the exposed cohort	Selection of the non-exposed cohort	Ascertainment of exposure	Outcome not present at baseline	Control for age and sex	Control for other confounding factors	Assessment of outcome	Enough long follow-up duration	Adequacy of follow-up of cohorts	Total
Hu 2021 (18)	1	1	1	1	1	1	1	0	1	8
Luo 2024 (19)	1	1	1	1	1	1	1	0	1	8
Sun 2024 (20)	1	1	1	1	1	1	1	1	1	9
Xiao 2025 (24)	0	1	1	1	1	1	1	0	1	7
Wu 2025 (23)	1	1	1	1	1	1	1	0	1	8
Su 2025 (22)	1	1	1	1	1	1	1	1	1	9
Li 2025 (21)	0	1	1	1	1	1	1	0	1	7

### Meta-analysis results

**Figure 2:**
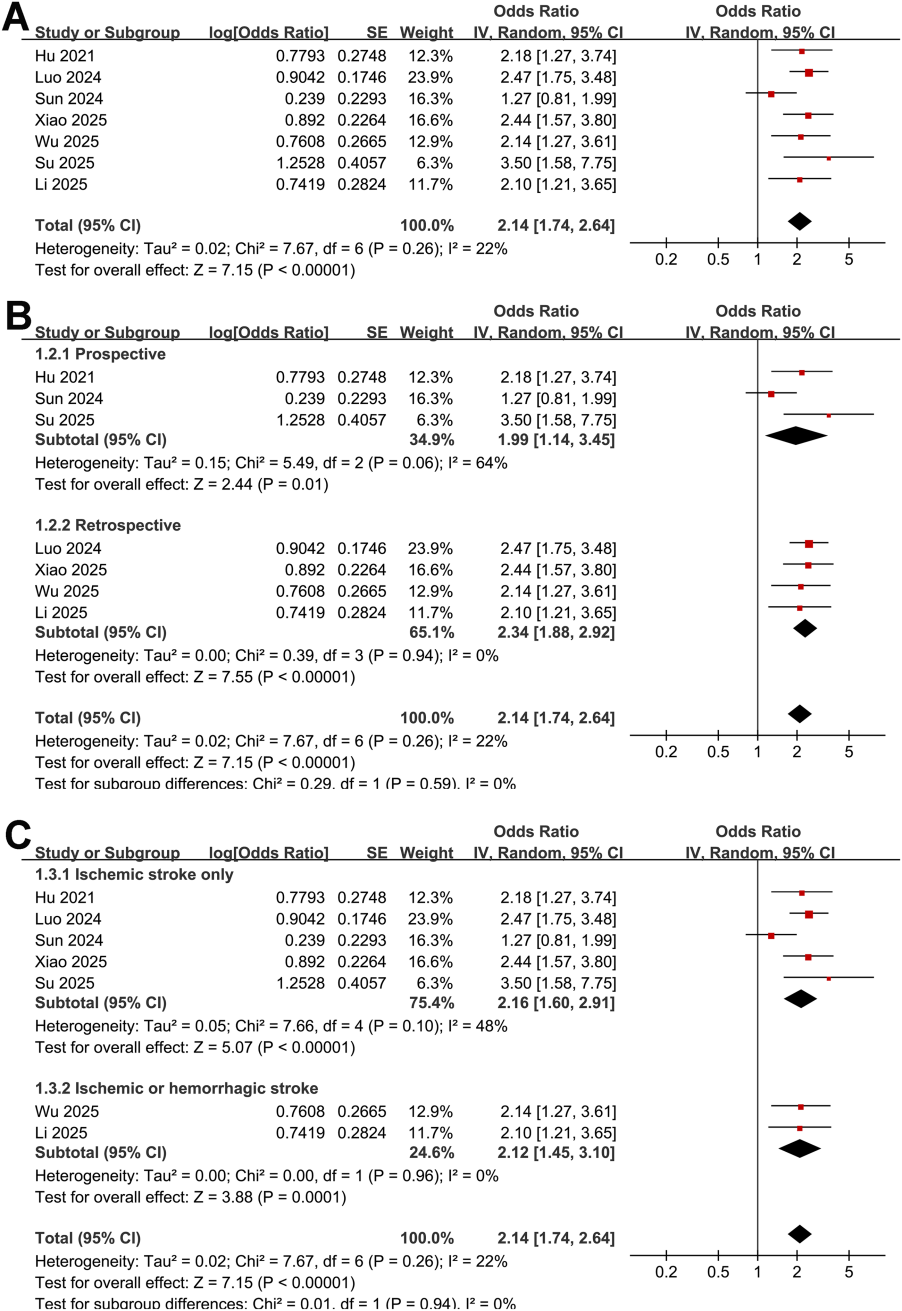
Forest plots showing the meta-analysis of the association between SII and PSD: **(A)** overall meta-analysis; **(B)** subgroup analysis by study design; **(C)** subgroup analysis by the diagnosis of the patients.

**Figure 3:**
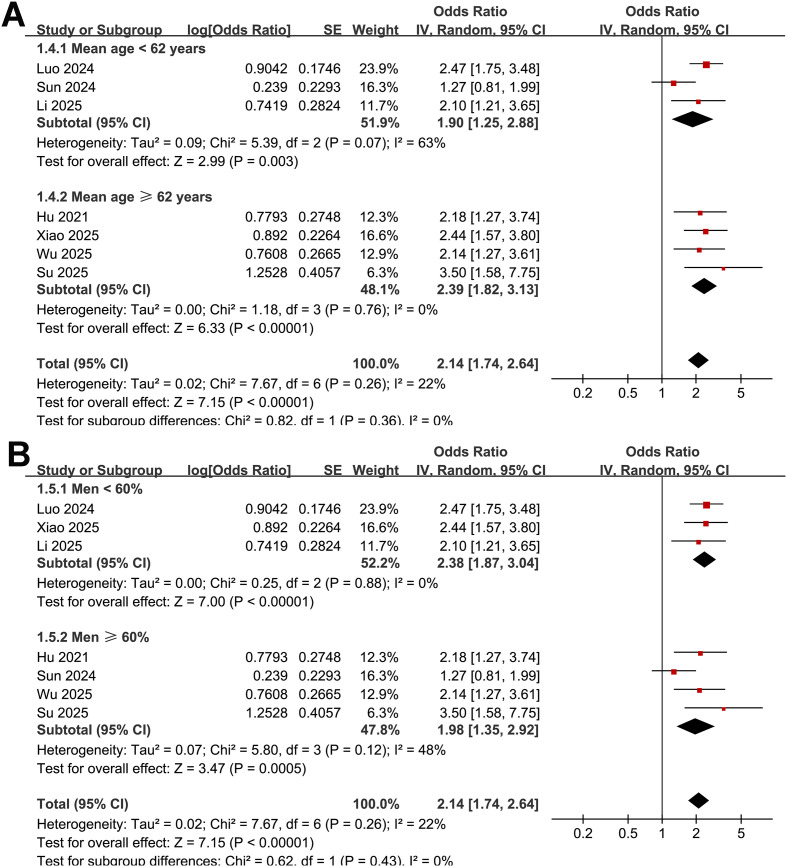
Forest plots for subgroup analyses of the association between SII and PSD: **(A)** stratified by mean age of the populations; **(B)** stratified by the proportions of men.

**Figure 4:**
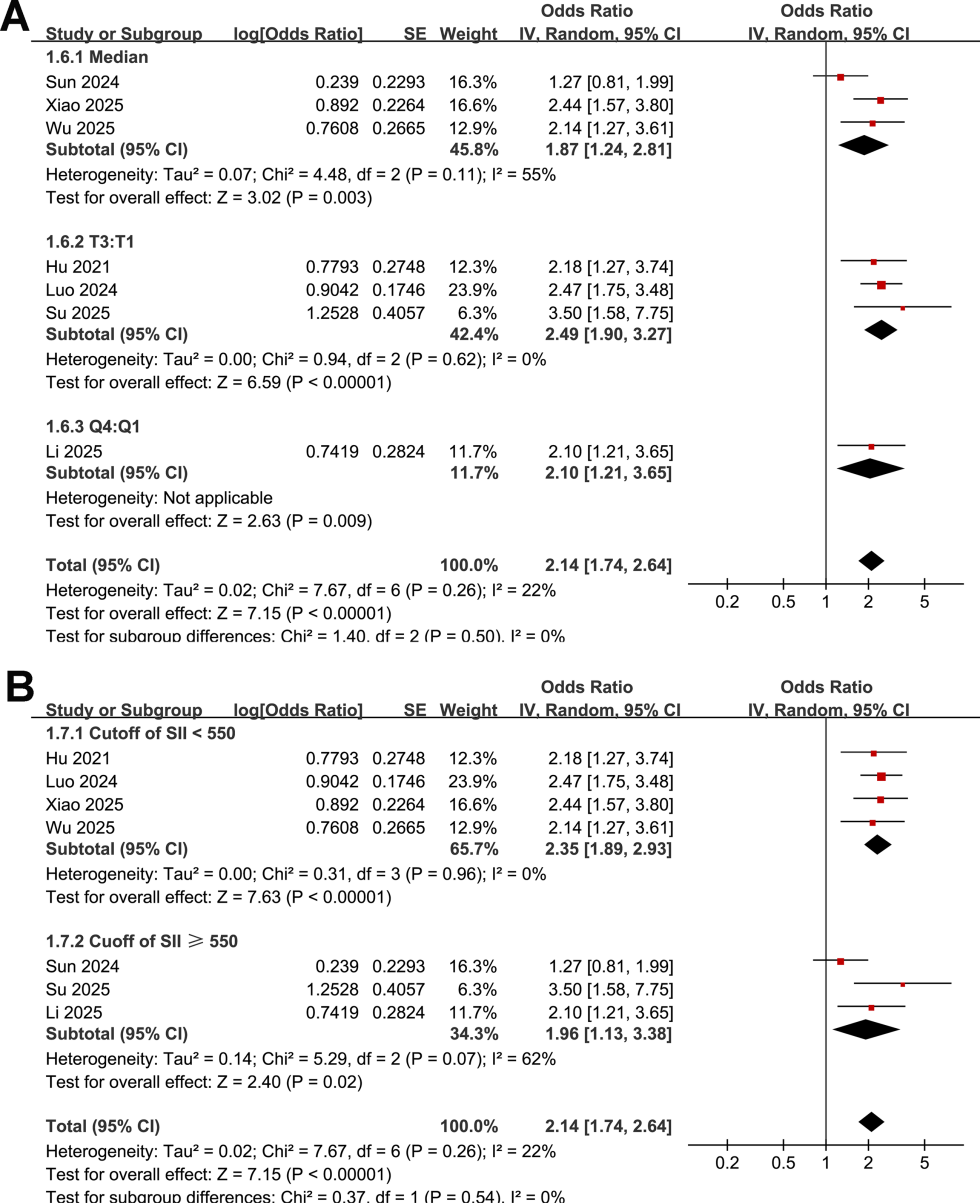
Forest plots for subgroup analyses of the association between SII and PSD: **(A)** stratified by the methods for determining the cutoffs of SII; **(B)** stratified by the cutoff values of SII.

**Figure 5:**
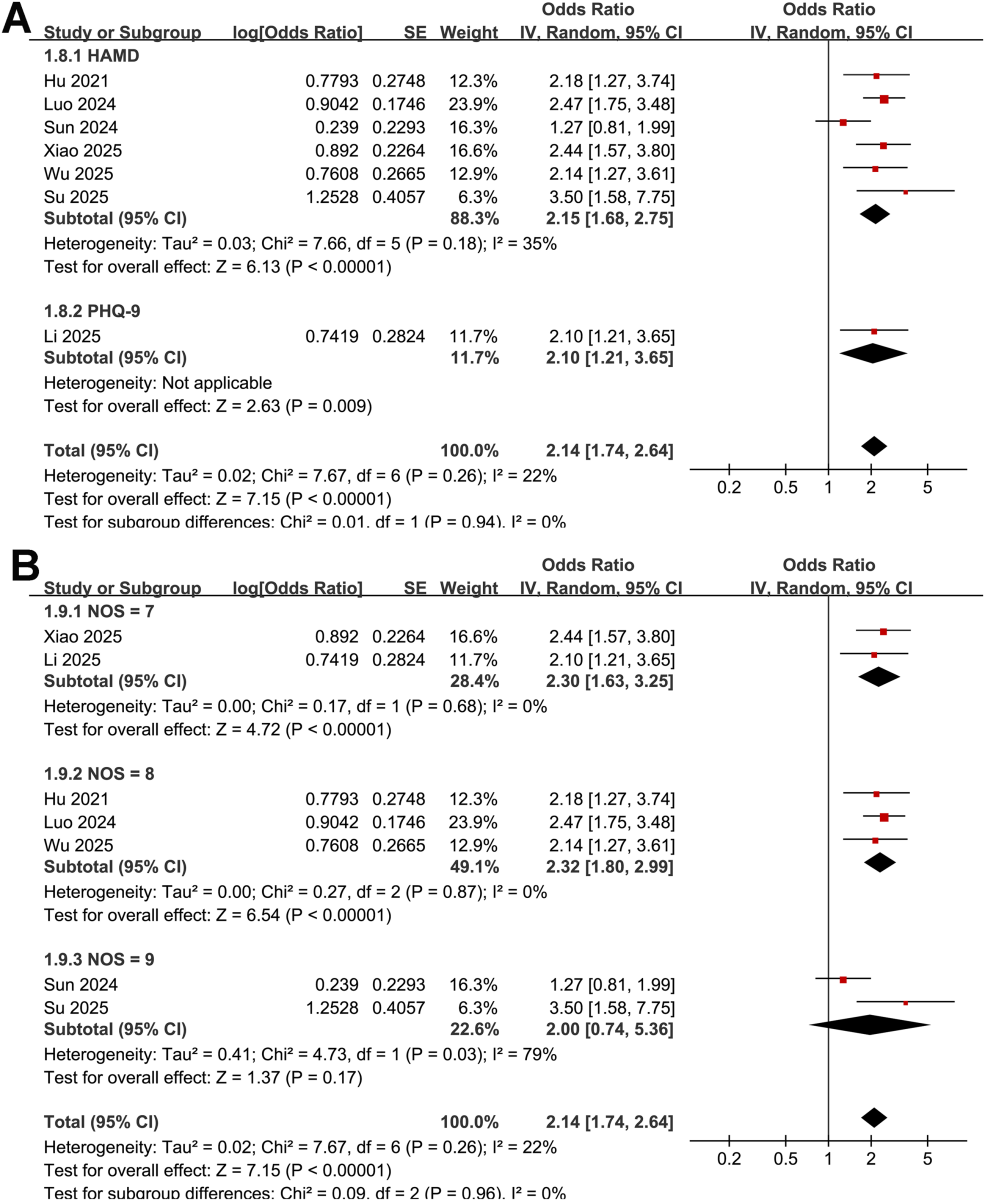
Forest plots for subgroup analyses of the association between SII and PSD: **(A)** stratified by methods for the diagnosis of PSD; **(B)** stratified according to the NOS scores.

The pooled analysis of the seven studies (18–24) demonstrated that a high SII at admission was significantly associated with an increased risk of PSD (OR: 2.14, 95% CI: 1.74–2.64, *p* < 0.001; **Figure 2A**) with low heterogeneity (Cochrane Q test *p* = 0.26; I² = 22%). Leave-one-out sensitivity analyses yielded consistent results, with pooled ORs ranging from 2.06 to 2.38 (all *p* < 0.05), indicating the robustness of the overall estimate. Notably, the sensitivity analysis excluding the study with the longest follow-up duration (12 months) (20) substantially reduced the heterogeneity (OR: 2.38, 95% CI: 1.95–2.89, *p* < 0.001; Cochrane Q test *p* = 0.92; I² = 0%), suggesting that differences in follow-up duration may partly contribute to between-study variability. Moreover, the sensitivity analysis pooling only studies reporting NIHSS-adjusted estimates (18–20, 22–24) also showed consistent results (OR: 2.15, 95% CI: 1.68–2.75, *p* < 0.001; Cochrane Q test *p* = 0.18; I² = 35%), indicating that the observed association was not solely explained by differences in baseline stroke severity at the study level. Further subgroup analysis showed consistent results in prospective and retrospective studies (OR: 1.99 vs. 2.34, *p* for subgroup difference = 0.59; **Figure 2B**), in studies with patients of ischemic stroke only and mixed stroke types (OR: 2.16 vs. 2.12, *p* for subgroup difference = 0.94; **Figure 2C**), in patients with the mean ages < 62 years and ≥ 62 years (OR: 1.90 vs. 2.39, *p* for subgroup difference = 0.36; **Figure 3A**), and in studies with the proportion of men < and ≥ 60% (OR: 2.38 vs. 1.98, *p* for subgroup difference = 0.43; **Figure 3B**). In addition, similar results were obtained for studies with cutoffs of SII defined by medians, tertiles, or quartiles (OR: 1.87 vs. 2.49 and 2.10, *p* for subgroup difference = 0.50; **Figure 4A**), and in studies with the cutoff values of SII < 550 and ≥ 550 (OR: 2.35 vs. 1.96, *p* for subgroup difference = 0.54; **Figure 4B**). Finally, the association between a higher SII and PSD was consistent in studies with PSD diagnosed by HAMD and PHQ-9 (OR: 2.15 vs. 2.10, *p* for subgroup difference = 0.94; **Figure 5A**), and in studies with the NOS of 7, 8, and 9 (OR: 2.30 vs. 2.32 and 2.00, *p* for subgroup difference = 0.96; **Figure 5B**).

### Publication bias

**Figure 6:**
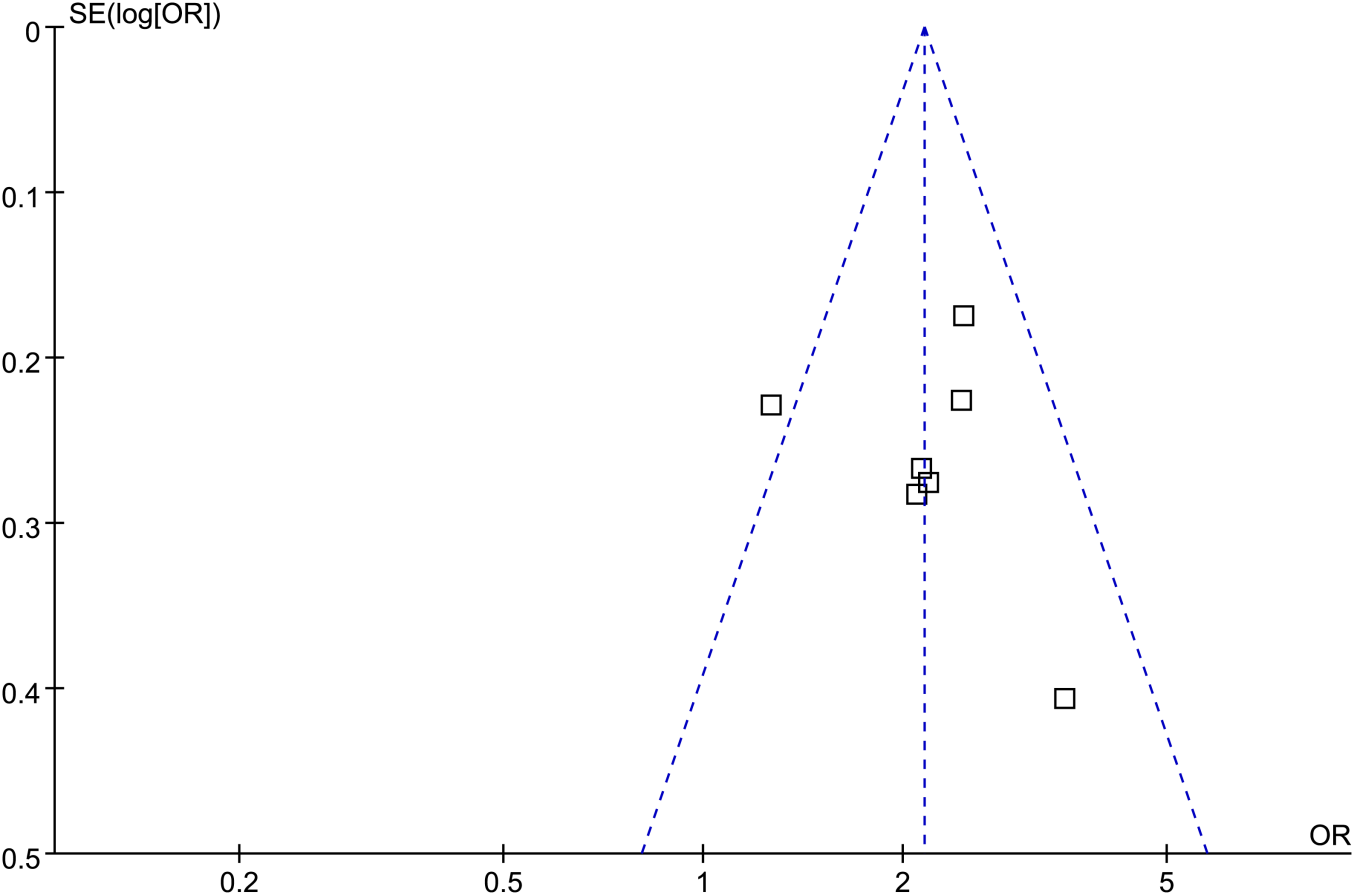
Funnel plots evaluating potential publication bias in the meta-analysis of the association between SII and PSD.

As shown in **Figure 6**, the funnel plots for the association between SII and PSD appeared largely symmetrical. Consistent with this, Egger’s test did not indicate statistically significant asymmetry (*p* = 0.44). However, since only seven studies were included, publication bias assessment should be interpreted cautiously.

## Discussion

In this systematic review and meta-analysis of 2,780 stroke patients from seven observational studies, elevated SII levels at admission were consistently associated with an increased risk of PSD. Across diverse clinical populations and settings, patients with higher SII exhibited approximately a twofold greater likelihood of developing depression after stroke compared with those with lower SII. This association remained robust across multiple sensitivity analyses and persisted in stratified analyses by study design, stroke type, SII cutoff approach, and depression assessment tool. These findings support SII as a promising inflammatory biomarker that may help identify stroke survivors at elevated risk for PSD and thereby inform early monitoring and preventative strategies.

Several biologically plausible mechanisms may underpin the observed association between elevated SII and PSD. SII is a composite index integrating the effects of three key leukocyte subtypes: neutrophils, lymphocytes, and platelets (31). Elevated neutrophil counts reflect enhanced innate immune activation and are a source of proinflammatory cytokines, such as interleukin-6 and tumor necrosis factor-α, which can cross or disrupt the blood–brain barrier and promote neuroinflammation (32, 33). Platelets contribute to inflammatory signaling through release of serotonin and other mediators that influence mood regulation and neuroplasticity (34, 35). In contrast, lymphopenia, which contributes to higher SII values, represents reduced adaptive immune regulation and diminished resilience against inflammatory stress (36, 37). Together, these components create a systemic environment characterized by heightened inflammation and impaired immune regulation. Chronic low-grade inflammation has been implicated in major depressive disorder through microglial activation, alteration of monoaminergic neurotransmission, dysregulation of the hypothalamic–pituitary–adrenal axis, and disruption of neurogenesis — pathophysiological processes that may also underlie PSD (38, 39). Thus, high SII may reflect a proinflammatory state that predisposes vulnerable stroke survivors to depressive symptomatology. However, it has to be mentioned that these inflammatory pathways are not specific to PSD and may also contribute to other neurological sequelae, such as cognitive impairment, fatigue, and functional decline. Therefore, the observed association between SII and PSD may reflect a broader vulnerability to adverse post-stroke outcomes rather than a depression-specific biological pathway. It is possible that depression preferentially manifests in some patients due to the sensitivity of mood-regulating neural circuits and neurotransmitter systems, particularly serotonergic and stress-response pathways, to inflammatory signaling. Nevertheless, this hypothesis remains speculative, and current evidence does not allow for definitive conclusions regarding outcome-specific mechanisms. Further mechanistic and longitudinal studies are required to clarify whether systemic inflammation differentially contributes to distinct post-stroke complications.

The consistency of findings across subgroups strengthens the plausibility of a true association. Sensitivity analyses excluding individual studies yielded pooled estimates that remained significantly elevated, indicating that no single study unduly influenced the overall effect. Moreover, an important observation is that the only study with a longer follow-up duration (12 months) (20) reported a non-significant association, whereas studies with shorter follow-up periods (typically within 3 months) consistently showed positive results. This pattern suggests that SII may primarily reflect acute peri-stroke inflammatory responses that are more strongly associated with early depressive symptoms, with the association potentially attenuating over time. However, this interpretation should be made cautiously, as only one study provided long-term follow-up data, and differences in patient characteristics, outcome definitions, and residual confounding may also contribute. Future longitudinal studies with repeated assessments are needed to clarify the temporal dynamics of the association between SII and PSD. In addition, subgroup analyses by stroke type (ischemic vs. mixed) showed similar magnitude of association, suggesting that the relationship between SII and PSD is not limited to a particular stroke subtype. Similarly, results were robust regardless of the method used to define SII thresholds, whether median-based, tertile, or quartile categorizations, and across different depression measures, including the HAMD and the PHQ-9. The absence of significant between-subgroup heterogeneity suggests that the association is broadly applicable across study designs and measurement approaches, reinforcing the potential generalizability of the findings. However, this finding of the subgroup analysis according to depression measures should be interpreted cautiously. The HAMD, which was used in most included studies, includes several somatic items that may overlap with post-stroke symptoms such as fatigue, sleep disturbance, and reduced physical activity (40, 41). This overlap may lead to overestimation of depressive symptoms in patients with more severe stroke-related impairment and represents a potential pathway through which residual confounding by stroke severity may persist. In contrast, the PHQ-9 focuses more on patient-reported symptoms and may be less influenced by clinician-rated somatic features (40, 41), but the limited number of studies using this tool precludes meaningful comparison. Therefore, the apparent consistency across assessment methods may partly reflect non-specific symptom detection rather than true equivalence in measuring PSD.

An additional consideration is that SII was measured exclusively at admission, whereas the timing of PSD assessment varied across studies, ranging from early (within weeks) to longer-term follow-up (up to 12 months). This temporal gap raises uncertainty regarding whether a single acute-phase inflammatory measurement can reliably reflect the biological processes underlying depression developing months later. It is possible that elevated SII at admission captures the intensity of the initial systemic inflammatory response to stroke, which may trigger or amplify downstream neuroinflammatory pathways and increase vulnerability to early depressive symptoms. However, SII may also act as a surrogate marker of stroke severity and related complications, which are themselves strong predictors of PSD. Therefore, the observed association may reflect a combination of direct inflammatory effects and indirect pathways related to disease severity. Future studies incorporating repeated measurements of inflammatory markers over time are needed to better delineate the temporal dynamics and causal relevance of SII in the development of PSD.

In a broader context, several inflammation-related biomarkers have been associated with PSD, including C-reactive protein, interleukin-6, and composite indices such as the neutrophil-to-lymphocyte ratio (NLR) (42). Compared with these markers, SII integrates multiple components of the immune response and may provide a more comprehensive reflection of systemic inflammatory status. However, SII is mathematically related to other indices such as the NLR, platelet-to-lymphocyte ratio, and monocyte-to-lymphocyte ratio, and therefore should be interpreted as part of a broader family of inflammation-based biomarkers rather than a fully independent construct. In the present study, SII was analyzed as a categorical variable because this was the predominant reporting format in the included literature, while continuous analyses were not feasible due to inconsistent data availability. These considerations support the biological plausibility of the findings while also highlighting the need for cautious interpretation and for future studies directly comparing different inflammatory markers and modeling approaches.

There are several methodological strengths of this meta-analysis. We conducted an up-to-date and comprehensive search of multiple English and Chinese databases, which minimized the risk of missing relevant studies. All included studies were observational designs with SII measured before the assessment of PSD, supporting a temporal relationship. Importantly, all studies reported multivariable-adjusted effect estimates, reducing the influence of confounding bias to the extent possible in non-randomized research. The use of a random-effects model accounted for potential residual heterogeneity, and rigorous sensitivity and subgroup analyses provided a thorough exploration of robustness and potential sources of variability. Despite these strengths, several limitations should be acknowledged when interpreting the results. First, the meta-analysis included studies of predominantly retrospective design, which may be prone to recall and selection biases (43). Although most included studies (18–20, 22–24) adjusted for stroke severity using NIHSS scores, the extent and consistency of adjustment varied, and NIHSS may not fully capture total ischemic burden or lesion characteristics. Therefore, residual confounding by stroke severity cannot be excluded. While sensitivity analysis restricted to NIHSS-adjusted estimates yielded consistent results, these findings should be interpreted cautiously. Future studies incorporating more comprehensive measures of stroke severity, such as infarct volume and lesion location, are needed to better clarify the independent role of SII. Notably, all included studies excluded patients with a history of pre-stroke psychiatric disorders, which may partially reduce confounding by prior depression or anxiety. However, the ascertainment of psychiatric history may have been incomplete or heterogeneous, and subclinical or undiagnosed conditions cannot be excluded. Therefore, residual confounding related to baseline psychological vulnerability remains possible and should be considered when interpreting the findings. Second, heterogeneity in stroke populations, patient demographics, comorbid conditions, healthcare settings, and timing of PSD assessment existed across studies. These differences may influence both SII values and depression risk but could not be fully explored due to lack of individual participant data (IPD) and variability in reporting. Third, although the association remained robust in sensitivity analyses, the observational nature of included studies precludes causal inference. Reverse causation — wherein factors associated with early depressive symptoms influence systemic inflammation — cannot be entirely ruled out. Fourth, while pooling SII as a categorical exposure facilitates clinical interpretation, the optimal cutoff value for predicting PSD remains unclear given the substantial heterogeneity in how “high SII” was defined across studies. Cutoff values varied widely and were derived using different approaches, including median splits, tertiles, quartiles, or study-specific thresholds. This variability limits comparability between studies and poses a challenge for clinical implementation, as no standardized or clinically validated threshold is currently available. In addition, most studies included patients with ischemic stroke only. The association between SII and PSD of patients with hemorrhagic stroke should be evaluated in future studies. Moreover, all included studies were conducted in China, predominantly in tertiary hospital settings, which may limit the generalizability of the findings. The risk and expression of PSD are influenced by a range of contextual factors, including healthcare systems, access to rehabilitation, cultural perceptions of mental health, social support structures, and socioeconomic conditions (44). These factors may affect both the assessment of depressive symptoms and the underlying relationship between inflammation and psychological outcomes. Therefore, the applicability of our findings to other populations remains uncertain. Future studies conducted in diverse geographic regions and healthcare settings are needed to validate the generalizability of the observed association. The results of the meta-analysis should be validated in prospective studies from other countries. Finally, although Egger’s test did not indicate significant publication bias, the small number of included studies limits the reliability of statistical tests for funnel plot asymmetry. Visual inspection suggested the presence of a study with a relatively large effect size and higher standard error, raising the possibility of small-study effects. Such effects are common in observational biomarker research and may reflect selective reporting, residual confounding, or differences in study size and methodological quality. Therefore, publication bias and small-study effects cannot be definitively excluded.

Clinically, these findings highlight the potential of SII as an accessible prognostic marker for PSD in routine practice. SII can be calculated from standard complete blood count parameters, which are routinely obtained in acute stroke care, making it an attractive candidate for risk stratification without additional cost or burden (45). Identifying patients at higher inflammatory burden early may prompt closer psychological monitoring, targeted support, or preventative interventions, such as psychosocial care or anti-inflammatory strategies, though such applications require prospective validation. Future research should aim to address the limitations of current evidence. Prospective, multicenter cohort studies with standardized SII measurement protocols, uniform PSD assessment timelines, and comprehensive adjustment for confounders are needed to confirm the temporal and potentially causal nature of the association. Future research should prioritize the standardization of SII cutoff values, ideally through large, prospective studies using receiver operating characteristic analyses or externally validated thresholds, to establish clinically meaningful and generalizable criteria for risk stratification. Exploration of the predictive performance of SII relative to and in combination with other biomarkers, as well as development of risk prediction models incorporating SII, would inform clinical utility. Mechanistic studies are also warranted to delineate the pathways linking systemic inflammation to post-stroke mood disorders and to identify potential intervention targets.

## Conclusions

In conclusion, this meta-analysis provides evidence that elevated SII is associated with an increased risk of PSD, suggesting that systemic inflammatory burden may play a role in post-stroke neuropsychiatric outcomes. While findings are compelling and robust across multiple analyses, they should be interpreted with caution given the observational design of underlying studies and residual methodological limitations. Further high-quality research is needed to validate the clinical utility of SII as a prognostic biomarker for PSD and to elucidate the underlying biological mechanisms.

## Data Availability

The original contributions presented in the study are included in the article/**Supplementary Material**. Further inquiries can be directed to the corresponding author.
