# Supplementary material for: The association between systemic immune-inflammation index and post-stroke depression: a meta-analysis
Source: Front Psychiatry. 2026 Jun 5;17:1817964. doi: 10.3389/fpsyt.2026.1817964 (PMC13279539; doi:10.3389/fpsyt.2026.1817964)
Supplement: Supplementary file 1 [file Table1.docx]

**Detailed search strategy for each database**

**PubMed**

("Systemic Immune-Inflammation Index"[Title/Abstract] OR "Systemic Immune-Inflammatory Index"[Title/Abstract] OR "Systemic Inflammation Index"[Title/Abstract] OR "Systemic-Immune-Inflammation Index"[Title/Abstract] OR SII[Title/Abstract]) AND ("Stroke"[Mesh] OR "Cerebral Infarction"[Mesh] OR "Brain Ischemia"[Mesh] OR "Intracranial Hemorrhages"[Mesh] OR stroke*[Title/Abstract] OR "cerebral infarction"[Title/Abstract] OR "cerebral hemorrhage"[Title/Abstract] OR "cerebrovascular accident*"[Title/Abstract] OR "intracranial hemorrhage"[Title/Abstract]) AND ("Depression"[Mesh] OR "Depressive Disorder"[Mesh] OR depression[Title/Abstract] OR depressive[Title/Abstract])

**Embase**

('systemic immune inflammation index'/exp OR 'systemic immune inflammation index':ti,ab OR 'systemic immune inflammatory index':ti,ab OR 'systemic inflammation index':ti,ab OR sii:ti,ab) AND ('stroke'/exp OR 'brain infarction'/exp OR 'brain hemorrhage'/exp OR stroke*:ti,ab OR 'cerebral infarction':ti,ab OR 'cerebral hemorrhage':ti,ab OR 'cerebrovascular accident*':ti,ab OR 'intracranial hemorrhage':ti,ab) AND ('depression'/exp OR 'depressive disorder'/exp OR depression:ti,ab OR depressive:ti,ab)

**Web of Science**

TS=(("systemic immune-inflammation index" OR "systemic immune-inflammatory index" OR "systemic inflammation index" OR "systemic-immune-inflammation index" OR SII)) AND TS=(stroke* OR "cerebral infarction" OR "cerebral hemorrhage" OR "cerebrovascular accident*" OR "intracranial hemorrhage") AND TS=(depression OR depressive)

**Wanfang**

("系统性免疫炎症指数" OR "系统免疫炎症指数" OR "SII") AND ("卒中" OR "脑卒中" OR "脑梗死" OR "脑出血" OR "脑血管意外") AND ("抑郁" OR "抑郁症")

**China National Knowledge Infrastructure (CNKI)**

SU = ("系统性免疫炎症指数" OR "系统免疫炎症指数" OR "SII") AND SU = ("卒中" OR "脑卒中" OR "脑梗死" OR "脑出血" OR "脑血管意外")

AND SU = ("抑郁" OR "抑郁症")
